# Recurrence pattern of non-syndromic familial congenital heart diseases among a large cohort of families from Egypt

**DOI:** 10.1186/s12887-022-03640-4

**Published:** 2022-10-19

**Authors:** Shaimaa Rakha, Rehab Mohy-Eldeen, Mohammad Al-Haggar, Mohammed Attia El-Bayoumi

**Affiliations:** 1 Pediatric Cardiology Unit, Pediatrics department, Faculty of Medicine, Mansoura University , El Gomhouria Street, 35516 Mansoura, Dakahlia Governorate Egypt; 2 Resident of Pediatrics, Mansoura University Children Hospital, Mansoura University, Mansoura, Egypt; 3 Genetics Unit, Pediatrics department, Faculty of Medicine, Mansoura University, Mansoura, Egypt; 4 Intensive care Unit, Pediatrics department, Faculty of Medicine, Mansoura University, Mansoura, Egypt

**Keywords:** Recurrence, Congenital Heart Diseases, Non-syndromic, Egyptian, Families

## Abstract

**Background:**

Congenital heart diseases (CHD) are the commonest congenital anomalies with increased risk in children born from families with affected members. However, various recurrence patterns of CHDs have been reported in different populations. Therefore, this work aimed to assess the recurrence patterns of CHDs in a large sample of Egyptian families.

**Methods:**

From January 2020 to October 2021, non-syndromic children with confirmed CHDs were recruited. Data were collected from guardians of the recruited children and hospital records, including the index case’s cardiac diagnosis and CHD diagnosis of other affected family members with to determine their recurrence pattern, consanguinity, and multi-gestation status.

**Results:**

A total of 130 recurrent cases with CHD were documented in 1960 families of children with CHD, including 66,989 members. Most recurrences were detected among first-degree relatives 50/130 (38.46%), especially siblings. Discordant recurrence was the most detected pattern (45.38%), followed by concordant recurrence (42.31%), and the least was group concordance. Recurrence rate was the highest for septal defects with left ventricular outflow tract obstruction (LVOTO) (11.8%) and anomalous venous drainage (11.1%), followed by septal defect with right ventricular outflow tract obstruction (RVOTO) (9.4%), isolated ventricular septal defect (VSD) category (8.2%) and LVOTO (8%). Familial recurrence was significant in consanguineous marriages [p = 0.0001; OR (95%CI) = 4.5 (2.25–9.01)] and in multi-gestations siblings: [p = 0.036; OR (95%CI) = 12.5(1.03–6.04)].

**Conclusion:**

The recurrence of non-syndromic CHD is evident among first-degree relatives in Egyptian families, with mostly a discordant recurrence pattern. Recurrence was more notable in septal defects with LVOTO, anomalous venous drainage, septal defect with RVOTO, isolated VSD, and isolated LVOTO diagnostic categories. This finding will significantly impact family counseling, emphasizing higher recurrence in consanguineous parents.

**Supplementary information:**

The online version contains supplementary material available at 10.1186/s12887-022-03640-4.

## Introduction

Congenital heart diseases (CHD) are the most common human congenital anomalies. The prevalence of CHD has increased substantially over time, from 0.6 to 1000 live births in 1930–1934 to progressively increasing in 2010–2017 to reach 9.410 in 1000 live births [1], which may be a result of improved diagnostic and screening modalities, including prenatal diagnosis [2]. Approximately one-third of patients with CHD are categorized as severe, requiring intervention in the first year of life [3]. Moreover, CHD is the most common cause of mortality associated with congenital defects in newborns [4]. It is often associated with fetal loss and contributes significantly to cardiovascular disease-associated disability [5, 6]. Recently in the US, 1 in 814 deaths was attributed to CHD in 1999–2017 [7].

The underlying etiology of CHD is complex and still not entirely understood [8]. Nora was the first to introduce the hypothesis of a multifactorial model in the sixties of the previous century, suggesting that several loci could interact together in association with environmental factors [9]. Since then, many environmental risk factors have been implicated in CHD development, such as maternal diabetes and teratogenic maternal medication [10]. Nevertheless, some families may exhibit a monogenic Mendelian pattern of CHD inheritance, while other cases of CHD are sporadic or exhibit non-Mendelian patterns without known environmental or genetic factors [11–15].

The risk of CHD is increased for children born into families affected by CHD, but variable rates have been reported among different populations and for various cardiac lesions [16–20]. Furthermore, patterns of familial recurrence of CHDs vary significantly among studies from different countries [21–23]. No previous research on a large group of families has assessed the recurrence pattern of CHDs in a Middle Eastern country like Egypt. Thus, research evaluating recurrence pattern among Egyptian families is warranted.

## Methods

This cross-sectional analysis included children affected with CHDs and their families recruited either from the cardiology outpatient clinics or admitted to the inpatient wards of a tertiary pediatric center from January 2020 to October 2021. Moreover, medical data regarding the details of cardiac diagnosis were retrieved from the patient records on the hospital information system. The institutional research board (IRB), Faculty of Medicine, Mansoura University, Egypt, approved this study. Additionally, the recruited participants provided consent to use their anonymous data.

### Inclusion criteria

Pediatric patients with confirmed CHDs diagnosed by at least an echocardiography at any time following birth were included and their families.

### Exclusion criteria

Patients with clinically suspected CHD, which is not fully verified using echocardiography, were excluded from the study. Additionally, patients with nationalities other than the Egyptians were omitted. Moreover, cases with incomplete information on the diagnosed lesion of other affected family members were excluded, and cases with a known other risk factor directly correlated with CHDs, such as maternal diabetes, drug intake. Other patients were excluded based on a confirmed diagnosis of chromosomal or genetic syndrome documented in hospital records or if suspected based on syndromic features especially in the presence of extracardiac anomalies. Suspected cases were reexamined by a geneticist in the work team. Finally, cases with incomplete or unconfirmed familial data about the recurrent diagnosis were excluded from further statistical analysis.

### Data collection

The primary recruited patient was described as ‘the index case’. Data about the index patients and their families were collected by detailed history taken from the patients᾽ parents, including the index case’s history of consanguinity between parents of the index case. The first cousins᾽ marriage was described as a third degree consanguineous marriage. In contrast, the first cousin once removed or double second cousin marriage was referred to as the fourth degree.

Moreover, whether the index patient resulted from singleton or multi-gestation pregnancies as twins, triplets, or more were assessed. The hospital information system was used to confirm age and retrieve the cardiac disease diagnosis for index patients and recurrent cases if available in the hospital system.

The primary cardiac lesion for the index patient was determined and classified into one of the main CHD phenotypic categories described by Botto et al. and further modified by Oyen et al. and Leirgul et al. [3, 18, 24]. The phenotypic groups were classified into: (1) isolated atrial septal defect (ASD), (2) isolated ventricular septal defect (VSD), (3) ASD and VSD, (4) atrioventricular septal defect (AVSD), (5) left ventricular outflow tract obstruction (LVOTO), (6) right ventricular outflow tract obstruction (RVOTO), (7) septal defect plus LVOTO, (8) septal defect plus RVOTO, (9) isolated patent ductus arteriosus (PDA) in infants born at term, (10) isolated PDA in preterm infants, (11) conotruncal heart defect, (12) conotruncal heart defect plus AVSD, (13) complex defects, (14) heterotaxia, (15) anomalous pulmonary venous return (APVR), and (16) other specified heart defects that do not fit into other groups, e.g. congenital mitral regurgitation. The category of unspecified CHD was excluded to increase the results accuracy through inclusion of confirmed cases only.

The confirmed diagnosis of CHDs in other affected family members was ascertained, and the family pedigree was studied to show the relationships between family members and patterns of inheritance. The relationship of that member to the index patient was categorized as follows; first-degree (parents and other siblings), second-degree (grandparents, grandchildren, uncles, aunts, nephews, nieces, and half-siblings), and third-degree (first cousins).

The concordance between the index case diagnosis and the recurrent family member with CHD was determined as suggested by Gill et al. [21]. Exact concordance was identified when the diagnosis in the other familial CHD case was identical to that seen in the index case, and group concordance if the defect belonged to the same phenotypic category of CHD. Otherwise, the recurrence was defined as discordant.

### Sample calculation

The sample size was calculated using Epi Info™ Statcalc (version 7.2.5.1., Centre for Disease Control and Prevention (CDC), Atlanta, Georgia, USA). ). We used a recurrence rate estimate of 5.33% based on a pilot study, with a confidence level of 95%, an error margin of 0.01%,, deriving a calculated sample size of at least 1935 to reach significance. A miniature study was performed as a pilot sample before carrying out the main research on 150 cases and their families through collecting data about presence or absence of CHD recurrence to calculate a preliminary the recurrence rate. These patients were not included in further analysis.

### Statistical analysis

Statistical analysis was performed using the Statistical Package for the Social Sciences (SPSS), version 25 (SPSS, Inc., an IBM Company, and Chicago, IL, USA). Descriptive statistics were used to describe the characteristics of the patients. Data were described using frequency and percentage. The association between variables was analyzed using the chi-square test. The result was considered significant when the probability value was less than 5% (p ≤ 0.05). The recurrence rate of CHD was calculated as a percentage of recurrent cases for the category of familial risk or CHD type in the index case [25].

## Results

The index cases that fulfilled the inclusion criteria were 1960, with available data to assess the familial pedigrees of CHD, which comprised 66,989 family members. Regarding index cases, the median age (interquartile range) was 48 (12–114) months, and 42 (2.14%) of index cases were of twins or triplets. A total of 207 cases were excluded from the study. Among them, 147 were subsequently diagnosed with syndrome, or association including: Down syndrome, Edward syndrome, Turner syndrome, Alagille syndrome, Williams’s syndrome, CHARGE syndrome, VACTREL, Ellisvan Creveled. Cutis Laxa, Marfan, Prune belly syndrome, DiGeorge Syndrome, and Crigler-Najjar syndrome. Furthermore, 21 patients were excluded due to having associated extracardiac birth defect (cleft lip/palate, hydronephrosis, polycystic kidney, hydrocephalus, dysmorphism, epilepsy, cerebral palsy, mental retardation). The other excluded cases were due to maternal comorbidities linked to CHD such as pregestational diabetes mellitus, rubella, and teratogenic drug intake during pregnancy.

Table 1 demonstrates the basic characteristics of familial recurrence in the families participated in the study. In the families under study, single recurrence was observed in 123 (97.6%), while two recurrences per family were in two families, and three recurrences per family were in only one family. Thus, the recurrent cases in the study were 130 cases in 126 families resulting in total recurrence rate of 6.43%(126/1960) in the studied families. The highest recurrence rate was 50/130(38.5%) among first-degree relatives, especially siblings 36/130 (27.69%) followed by maternal CHD 9/130 (6.92%). Among 1780 families with at least a sibling in addition to the index case, sibling recurrence was detected in 1.9% of families.

**Table 1:** Familial Recurrence in the Study Families

Variables	Families N (%)
Frequency of recurrent cases per family :	
• One recurrence	123/126 (97.62%)
• Two recurrences	2/126 (1.59%)
• Three recurrences	1/126 (0.79%)
Number of recurrent cases in the study :	130
Familial relation between recurrent cases and index case:	
First-degree relatives	50/130 (38.46%)
• Maternal	9/130 (6.92%)
• Paternal	5/130 (3.85%)
• Siblings	36 /130(27.69%)
Second-degree relatives	32/130 (24.62%)
Third-degree relatives (Cousins)	48/130 (36.92%)
Concordance of familial recurrence	
• Exact concordant recurrence	55/130 (42.31%)
• Group concordant recurrence	16/130(12.31%)
• Discordant recurrence	59/130 (45.38%)

**Fig. 1:**
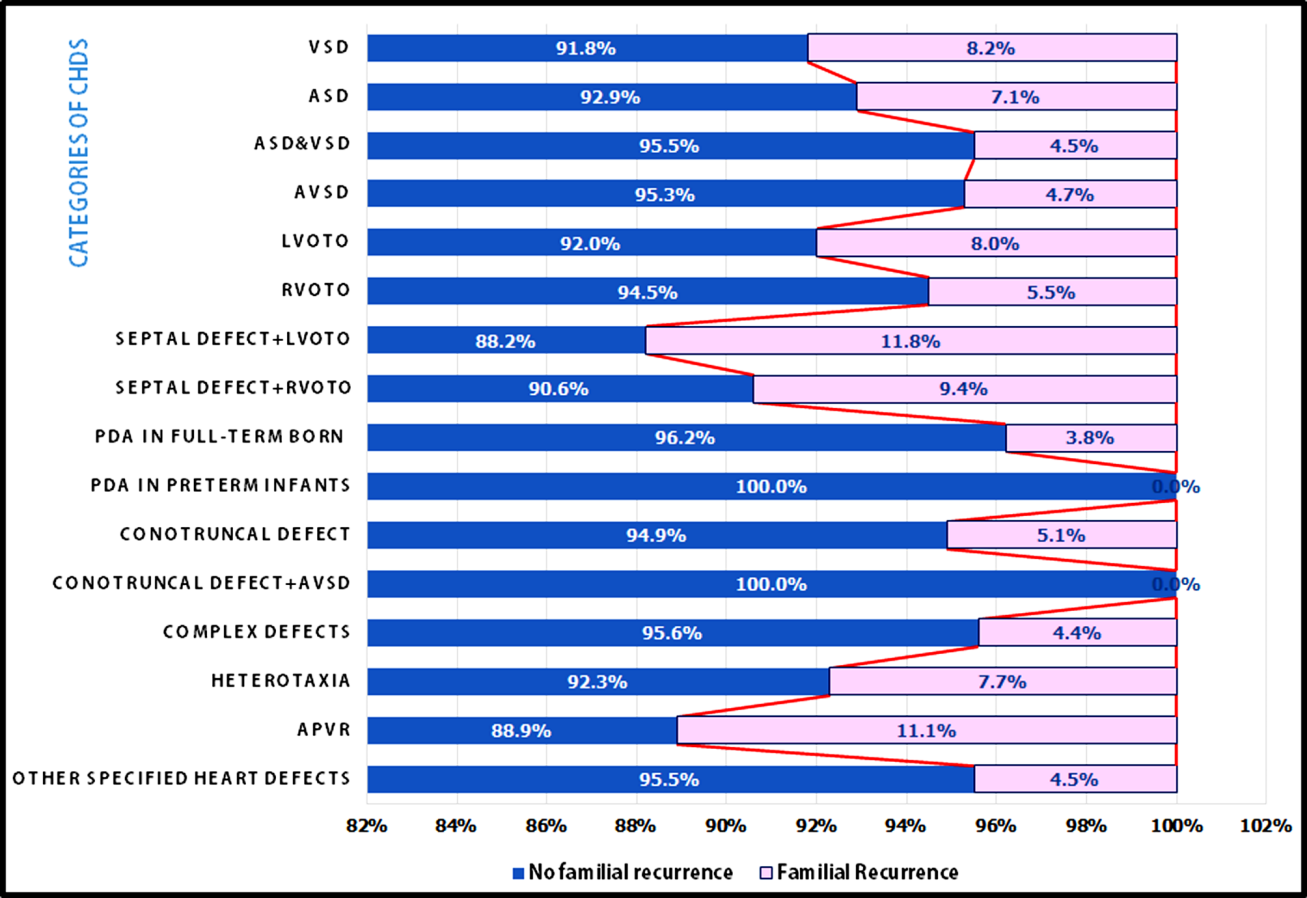
Familial recurrence vs. non-recurrence percentage of families in each diagnostic category of CHDs. **APVR**: anomalous pulmonary venous return, **ASD**: atrial septal defect, **AVSD**: atrioventricular septal defect, **LVOTO**: left ventricular outflow tract obstruction, **PDA**: patent ductus arteriosus, **RVOTO**: right ventricular outflow tract obstruction, **VSD**: ventricular septal defect

In Table 2, the recurrence rate of each diagnostic category of CHD and its concordance are presented. The most frequent diagnostic category of index cases was isolated VSD 490/1960 (25%) of patients, followed by isolated ASD 337 (17.2%) then RVOTO (13.8%). Notably, the highest recurrence rate was in the category of septal defect plus LVOTO (11.8%), followed by anomalous pulmonary veins (11.1%), then septal defect plus RVOTO (9.4%), isolated VSD (8.2%), and isolated LVOTO (8%), as indicated in Fig. 1. However, no recurrence was documented in the conotruncal plus AVSD or PDA in the preterm group.

**Table 2:** Recurrence rate and concordance for each diagnostic classification of CHDs of the index case

Phenotypic category of Index case CHD	Total Study Families N = 1960	Families without recurrence N = 1834		Families with recurrence N = 126	Concordance of Recurrence N = 130 cases		
					**Exact concordant**	**Group concordant**	**Discordant**
Isolated VSD	490 (25.0%)	450 (91.8%)	40 (8.2%)		23 (57.5%)	2 (5.0%)	15 (37.5%)
Isolated ASD	337 (17.2%)	313 (92.9%)	24 (7.1%)		12 (50.0%)	4 (16.7%)	8 (33.3%)
ASD&VSD	44 (2.2%)	42 (95.5%)	2 (4.5%)		0 (0%)	1 (50.0%)	1 (50.0%)
AVSD	43 (2.2%)	41 (95.3%)	2 (4.7%)		0 (0%)	1 (50%)	1 (50%)
LVOTO	138 (7.0%)	127 (92.0%)	11 (8.0%)		7 (63.6%)	1 (9.1%)	3 (27.3%)
RVOTO	271 (13.8%)	256 (94.5%)	15 (5.5%)		6 (37.5%)	0 (0%)	10 (62.5%)
Septal defect plus LVOTO	17 (0.9%)	15 (88.2%)	2 (11.8% )		0 (0%)	2 (100%)	0 (0%)
Septal defect plus RVOTO	32 (1.6%)	29 (90.6%)	3 (9.4%)		0 (0%)	1 (33.3%)	2 (66.7%)
Isolated PDA in infants born at term	235 (12.0%)	226 (96.2%)	9 (3.8%)		1 (11.1%)	0 (0%)	8 (88.9%)
Isolated PDA in preterm infants	2 (0.1%)	2 (100%)	0 (0%)		0 (0%)	0 (0%)	0 (0%)
Conotruncal	215 (11%)	204 (94.9%)	11(5.1%)		5(35.7%)	2(14.3%)	7(50%)
Conotruncal plus AVSD	2 (0.1%)	2 (100%)	0 (0%)		0 (0%)	0 (0%)	0 (0%)
Complex defects	90 (4.6%)	86 (95.6%)	4 (4.4%)		0 (0%)	2 (50.0%)	2 (50.0%)
Heterotaxia	13 (0.7%)	12 (92.3%)	1(7.7%)		0 (0%)	0 (0%)	1(100%)
APVR	9 (0.5%)	8 (88.9%)	1 (11.1%)		0 (0%)	0 (0%)	1 (100%)
Other specified heart defect	22 (1.1%)	21 (95.5%)	1 (4.5%)		1 (100%)	0 (0%)	0 (0%)

**APVR**: anomalous pulmonary venous return, **ASD**: atrial septal defect, **AVSD**: atrioventricular septal defect, **LVOTO**: left ventricular out flow tract obstruction, **PDA** :patent ductus arteriosus, **RVOTO**: right ventricular out flow tract obstruction, **VSD**: ventricular septal defect

**Fig. 2:**
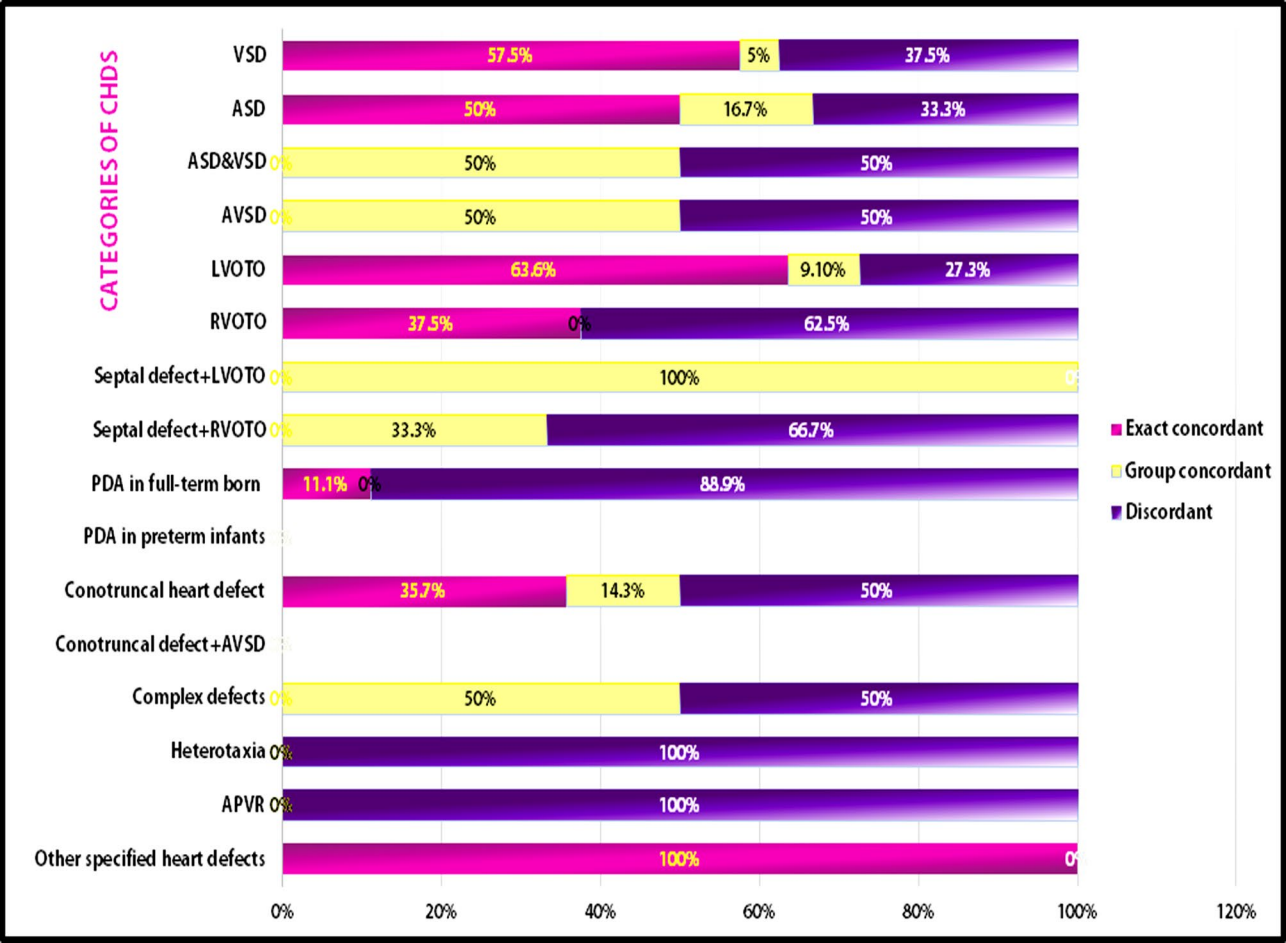
Concordance of familial Recurrence for diagnostic categories of CHDs. **APVR**: anomalous pulmonary venous return, **ASD**: atrial septal defect, **AVSD**: atrioventricular septal defect, **LVOTO**: left ventricular outflow tract obstruction, **PDA**: patent ductus arteriosus, **RVOTO**: right ventricular outflow tract obstruction, **VSD**: ventricular septal defect

The rate of each concordance pattern of familial recurrence for each diagnostic category of CHDs is illustrated in Fig. 2. The exact concordance of recurrence varied from 0 to 100% with 100% recurrence in the category of other specified CHD, which was only one case, then LVOTO category (63.6%), followed by isolated VSD (57.5%) and isolated ASD (50%). For group concordance, the highest rate was 100% in the category of septal defect plus LVOTO, followed by ASD&VSD in 50% of recurrent cases. Discordant recurrence was evident in heterotaxy and anomalous pulmonary veins (100%), followed by isolated PDA in the term-born infant’s category in 88.9%. The supplementary material demonstrates examples of pedigrees for some families with confirmed recurrent CHD.

In Table 3, the prevalence of CHD for each degree of family members in the study is demonstrated. The highest prevalence was observed in first-degree relatives, reaching (20.87%) followed by second-degree (8.52%).

**Table 3:** CHD prevalence in each studied degree of family members

	At risk members (families without R)	At risk members (families with R)	CHD cases (families with R) (Index + R)	CHD Prevalence
First degree	7127	493	(1960 + 50)	20.87%
Second degree	19,975	1422	(1960 + 32)	8.52%
Third degree	33,527	2355	(1960 + 48)	5.30%
total	60,629	4270	(1960 + 130)	3.12%

**CHD**: congenital heart diseases, **R**: recurrence.

The prevalence of consanguinity among parents of the index patients with CHD in our study was 343/1960 (17.5%) with 261/343 (76.1%) third-degree related parents and 18/343 (5.2%) fourth-degree related parents. The recurrence rate in siblings of consanguineous parents was 16/318(5.03%). The multi-gestation sibling for index cases were documented in 42/1960 (2.1%) of index patients, 12 (28.6%) monozygotic twins, 28 (66.7%) dizygotic twins, and 2 (4.7%) triplet. The recurrence rate of CHD in a multi-gestation sibling was detected in 6/42 (14.3%) of families with 4/6(66.6%) in monozygotic twins, 1/6(16.7%) Dizygotic twins, 1/6 (16.7%) Triplet.

Table 4 demonstrates the relationship between multi-gestation and consanguinity with the recurrence rate of CHDs. A significant recurrence rate was detected in siblings of consanguineous marriages in relation to non-consanguineous (16/302, 5% vs. 17/1445, 1.2% respectively) with P = 0.0001; OR (95%CI): 4.5 (2.25–9.01). Similarly, statistically significant familial recurrence was detected in multi-gestational siblings P = 0.036; OR (95%CI):12.5 (1.03–6.04).

**Table 4:** Relation between consanguinity, multi-gestational siblings and recurrence risk

	Familial recurrence		p-value	OR (95% CI)
	**Recurrence**	**No recurrence**		
Consanguinity**				
• Consanguineous parents	16 (5%)	302 (95%)	0.0001*	4.5 (2.25–9.01)
• Non-consanguineous parents	17 (1.2%)	1445 (98.8%)		
Multi-gestational pregnancy				
• Multi-gestational siblings	6 (14.3%)	36 (85.7%)	0.036*	2.5 (1.03–6.04)
• Singletons	120 (6.3%)	1798 (93.7%)		

**OR**: odds ratio, **CI**: confidence interval

*Significant p-value less than 0.05

** In parents with other offspring than the index case (1780 family)

Gender and parental consanguinity of index patients is for each category of CHD with sibling recurrence patterns are presented in Table 5. The highest percentage of consanguinity was detected in index patients with anomalous pulmonary veins (33.3%) and heterotaxia (30.8%). At the same time, three categories did not include index patients with parental consanguinity, which were PDA in preterm, conotruncal plus VSD and other specified heart defect. Exact concordance was the most frequent recurrence pattern in sibling recurrence whatever the consanguinity status; however, ASD&VSD, concotruncal and complex lesions have 100% group concordance pattern in recurrent siblings of consanguineous parents.

**Table 5:** Gender ratio and parental consanguinity for index patients in each CHD category and sibling pattern of recurrence for status of parents consanguinity

Category of CHD	Gender M: F Ratio	Consanguinity*	Concordance of sibling recurrence	N(%)**
Isolated VSD	1.19	Non-consanguineous 397(81)	Exact concordant	2 (66.67)
			Discordant	1(33.33)
		Consanguineous 93(19)	Exact concordant	4 (66.7)
			Group concordant	1 (16.7)
			Discordant	1 (16.7)
Isolated ASD	1.14	Non-consanguineous 276(81.9)	Exact concordant	1 (50)
			Discordant	1 (50)
		Consanguineous 61(18.1)	Exact concordant	1 (100)
ASD&VSD	0.63	Non-consanguineous 36(81.8)	-	-
		Consanguineous 8(18.2)	Group concordant	1 (100)
AVSD	0.65	Non-consanguineous 40(93)	-	-
		Consanguineous 3(7)	-	-
LVOTO	2.29	Non-consanguineous 115(83.3)	Exact concordant	3 (100)
		Consanguineous 23(16.7)	Exact concordant	1 (50)
			Discordant	1 (50)
RVOTO	1.08	Non-consanguineous 228(84.1)	Exact concordant	1(33.33)
			Discordant	2 (66.67)
		Consanguineous 43(15.9)	Exact concordant	2 (100)
Septal defect plus LVOTO	1.13	Non-consanguineous 13(76.5)	-	-
		Consanguineous 4(23.5)	-	-
Septal defect plus RVOTO	1.67	Non-consanguineous 27(84.4)	Group concordant	1(100)
		Consanguineous 5(15.6)	-	-
Isolated PDA among infants born at term	0.73	Non-consanguineous 195(83)	Exact concordant	1(33.33)
			Discordant	2 (66.67)
		Consanguineous 40(17)	Discordant	2 (100)
			-	-
Isolated PDA in preterm infants	^-all males^	Non-consanguineous 2(100)	-	-
Conotruncal heart defect	1.17	Non-consanguineous 176(81.9)	Exact concordant	2(100)
		Consanguineous 39(18.1)	Group concordant	1(100)
Conotruncal heart defect plus AVSD	1.00	Non-consanguineous 2(100)	-	-
Complex defects	0.70	Non-consanguineous 73(81.1)	-	-
		Consanguineous 17(18.9)	Group concordant	1(100)
Heterotaxia	1.60	Non-consanguineous 9(69.2)	-	-
		Consanguineous 4(30.8)	-	-
APVR	0.80	Non-consanguineous 6(66.7)	-	-
		Consanguineous 3(33.3)	-	-
			-	-
All other specified heart defect	0.69	Non-consanguineous 22(100)	-	-
			-	-

**APVR**: anomalous pulmonary venous return, **ASD**: atrial septal defect, **AVSD**: atrioventricular septal defect, **LVOTO**: left ventricular out flow tract obstruction, **M: F Ratio**: male: female ratio, **PDA**: patent ductus arteriosus, **RVOTO**: right ventricular out flow tract obstruction, **VSD**: ventricular septal defect. - : empty cell means no recurrence

*Data are presented as number (percentage)

** Total Families with sibling recurrences (33 family); One family has 2 sib recurrence, One family has 3 sib recurrence (Total of 36 siblings recurrences)

## Discussion

This cross-sectional study included 1960 children with CHD and data about 66,980 family members comprising 130 recurrent cases. Most recurrences were detected among first-degree relatives 50/130 (38.46%), especially siblings. Discordant recurrence was the most detected pattern (45.38%), followed by concordant recurrence (42.31%), and the least was group concordance. The recurrence rate was the highest for septal defects with LVOTO category (11.8%) and anomalous venous drainage (11.1%), followed by septal defect plus RVOTO (9.4%), isolated VSD category (8.2%) and LVOTO (8%). Familial recurrence was significant in consanguineous marriages p = 0.002; OR (95%CI) = 1.91(1.27–2.87) and in multi-gestational siblings: p = 0.036; OR (95%CI) = 12.5(1.03–6.04).

Information about the probability of familial CHD recurrence is essential for the family counselling process. However, the lack of a large-scale study in any Middle Eastern country about the CHD recurrence pattern mandates the current work. Thus, the familial recurrence of CHDs was studied, emphasizing the significant impact of consanguinity and multi-gestation.

The highest recurrence rate in our study is in septal defects with LVOTO category (11.8%) and anomalous venous drainage (11.1%), followed by septal defect with RVOTO . In a UK hospital-based study on 727 adults with CHDs, the highest recurrence was in AVSD (10%), followed by (Tetralogy of Fallot) TOF (3%) [26]. A registry-based study on the Danish population showed the highest recurrence risk for heterotaxia and AVSD after the exclusion of chromosomal aberrations [18]. Using fetal echocardiography in a study from Norway, Gill et al. found a recurrence rate of 80% for isolated AVSD and 64% for laterality defects [21]. While in Indonesian families, PDA was the most recurring lesion with no consanguinity history in any of their included families with confirmed recurrence [27].

Regarding the recurrence pattern, discordant recurrence (45.38%) was the most detected in the current work, with slightly lower exact recurrence (42.31%), while Ellesøe et al. documented a higher degree of discordance, reaching twice the possibility of concordance. Moreover, they suggested that co-occurrence of discordant heart defects follows distinct pattern, which suggests an underlying developmental mechanism, such as sharing susceptibility genes. Nevertheless, a higher degree of exact concordance was observed in specific categories such as isolated VSD, isolated ASD, coarctation of the aorta, PDA, and TOF [23]. These results are consistent with our findings as exact concordance was predominantly detected in 63.6% of the LVOTO category as in COA and aortic stenosis cases, followed by 57% exact recurrence in isolated VSD. Similarly, discordant recurrence was the most detected pattern in 51.5% of recurrences detected by fetal echocardiography [25].While Gill et al. found that group concordance was more frequent in 44% of their included families, exact concordance was in 55% of families with two or more recurrences, and the exact concordance was higher in AVSD and laterality defects [21]. On the contrary, in Denmark, Oyen et al. found that similar recurrence in first-degree relatives was the most predominant; nevertheless, dissimilar recurrences were weak [18].

It was suggested that a single-gene disorder could be the etiologic factor in the recurrence of some non-syndromic heart defects especially isolated ASD, isolated AVSD, LVOTO, and anomalous pulmonary venous drainage [28–32]. These isolated heart defects are proposed to be inherited in some families with reduced penetrance. Shared undetermined environmental factors in successive pregnancies could also underlie the increased relative risk.

Recurrence was mainly detected among first-degree relatives in this study. Similarly, Oyen et al. found that the recurrence risk for similar lesions was the highest among first-degree relatives [18]. In the current study, we have a higher recurrence rate in children of affected mothers with CHD more than affected fathers. Whittemore et al. reported a higher recurrence rate in the offspring of mothers with CHD (16.1%) [33]. Also, Burn et al. reported a higher recurrence rate for children of affected mothers compared to the cases with an affected father (4.1% and 2.2%, resp.) [26]. They suggested an imprinting pattern could theoretically explain the excess of cardiac defects where the phenotypic effect depends on the expression of the maternally inherited allele as deleterious maternal alleles would have more harmful effect than paternally originated allele. The preponderance of affected offspring of mothers with CHD may be partly explained by cytoplasmatic inheritance, which is a transmission of maternal cytopathy [25]. Recently, Øyen et al. found a significant maternal-to-paternal recurrence ratio of 1.82 (P < 0.0001), and the maternal excess was more pronounced for same-phenotype recurrence particularly conotruncal and LVOTO defects. They explained the maternal effect on recurrence through the threshold liability hypothesis that was initially proposed as an explanation for the familial recurrence of pyloric stenosis, in which the less affected sex is presumed to have a higher genetic burden; therefore, leading to a more substantial risk of parent-offspring recurrence for parents of that sex [34].

Our study found a recurrence risk of 1.9% in families with siblings, but 52% were exact recurrences. A registry-based study from Sweden; recurrence risk in siblings was 1.26% (12/1114) [35]. However, Brodwall et al. found a higher CHD recurrence rate among full siblings (4.1%), with more discordant recurrence in 54% [36]. Increased risk in siblings of children with CHD could be explained by the shared genetic factors, shared environment, or a combination of both as one single strong risk factor could be the cause in some families or aggregation of multiple low-risk factors of CHD for other families [36, 37]. This could be further proved by a statistically significant increased recurrence risk in multi-gestational siblings as in our study, which is consistent to findings from previous studies [36–39].

Consanguinity has an established relationship with increased risk of CHD in population-based and case-control studies [41–42]. Consanguineous mating, is a widely accepted in Arab countries. Consequently, a significant genetic implication on the offspring of consanguineous parents as it renders the genomes of the offspring autozygous due to the identically inherited chromosomal segments from both parents resulting in frequent autosomal recessive diseases in the offspring [43]. Moreover, consanguinity rates vary between different types of CHD with the highest incidences in ASD, TOF, and valvular aortic stenosis (AS) in a series, supporting the theory that these defects may be caused by recessive genes [44]. In contrast, the highest consanguinity rates among our index cases were detected in anomalous venous return and heterotaxia. The highest consanguinity rates especially in these diagnostic groups could be due to the population differences which requires further confirmation investigating the genetic etiologies or may be partly due to the insufficient cases included within these groups in the current work. This work detected a significantly higher recurrence rate of CHD among the offspring of consanguineous marriage than that in non-consanguineous parents with a previous child with CHD. ASD&VSD, concotruncal and complex lesions have 100% group concordance in recurrent offspring of consanguineous parents.

One of the strengths of the current study is the significant sample size calculated with the exclusion of families with incomplete or unsure information. Moreover, the limited pregnancy terminations among the Egyptian population due to cultural and religious backgrounds result in more reliable recurrence figures for CHD.

The drawback of our study is that most retrieved information depended on the guardians of the index patient regarding their knowledge and communication with other family members. It was not possible to access medical records of older family members who were not in our hospital system. Still, at least detailed echocardiography confirming diagnosis was required to include these cases. The challenges we have encountered in the current work elucidate the need and value of a nationwide registry for CHDs to tackle the effect of different familial and environmental factors on the pattern of CHD recurrence. Furthermore, future studies will be required to address the genetic background of Egyptian families with a recurrence of CHD.

## Conclusion

The recurrence rate of non-syndromic CHD among Egyptian families is evident among first-degree relatives. Discordant recurrence was the most detected pattern of recurrence. The highest recurrence percentage was found in septal defects with LVOTO and anomalous pulmonary venous drainage, followed by septal defect with RVOTO,  isolated VSD and isolated LVOTO categories. This finding will significantly impact family counseling process, especially the significant high recurrence in relation to consanguinity.

## Electronic supplementary material

Below is the link to the electronic supplementary material.

Supplementary Material 1

## Data Availability

The data are not publicly available because they are containing information that could compromise the privacy of research participants in this study. Data are available from the corresponding author on reasonable request.
