# Supplementary material for: Recurrence pattern of non-syndromic familial congenital heart diseases among a large cohort of families from Egypt
Source: BMC Pediatr. 2022 Oct 19;22:607. doi: 10.1186/s12887-022-03640-4 (PMC9580194; doi:10.1186/s12887-022-03640-4)
Supplement: Supplementary file 1 — Supplementary Material 1 [file 12887_2022_3640_MOESM1_ESM.docx]

**Supplementary Material**:


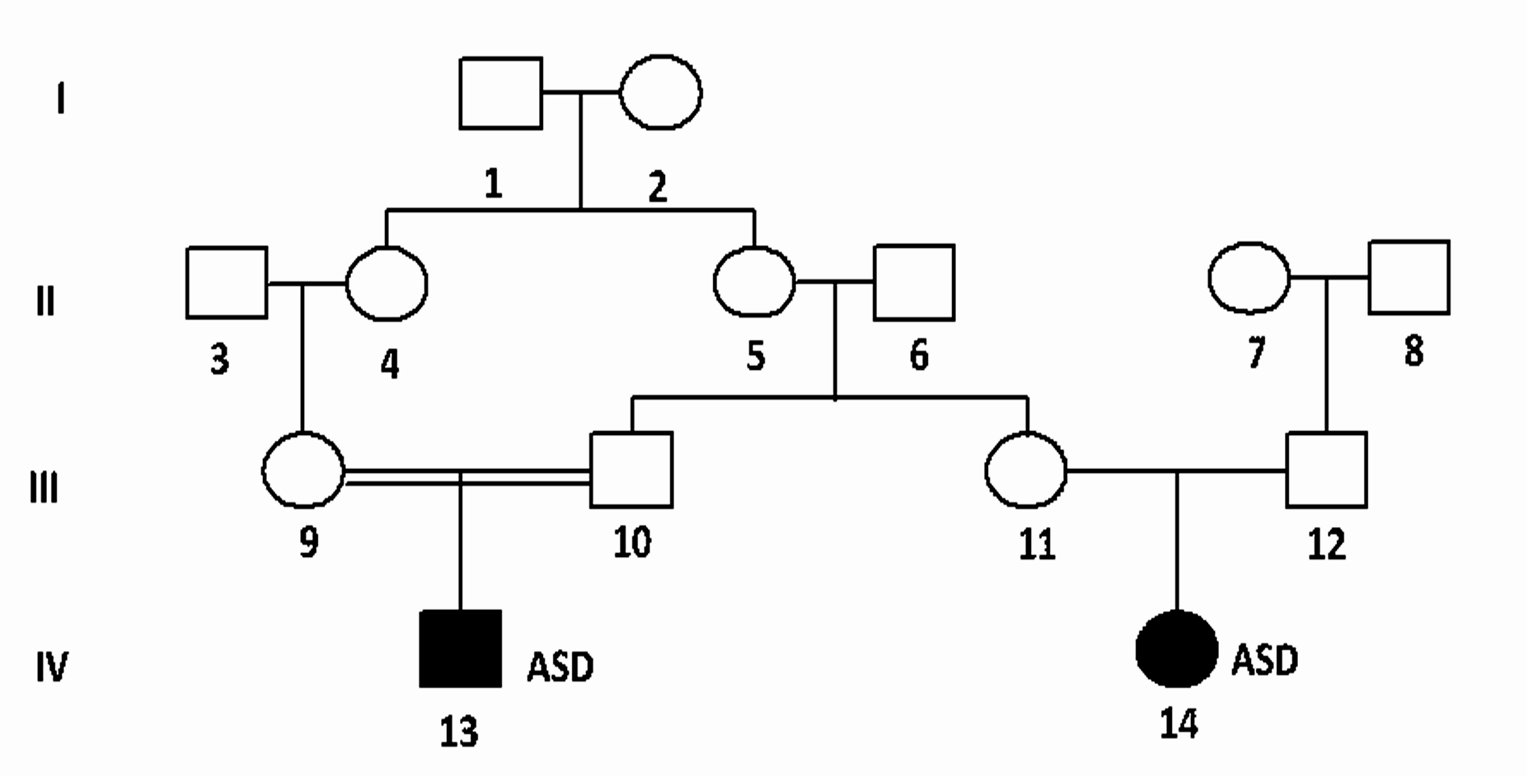


**Figure 3:** A Pedigree showing exact concordant recurrence of large secondum ASD in 2 cousins (IV 14, 13); IV.13 from consanguineous marriage between III.9, III.10.





**Figure 4:** A pedigree showing exact concordant recurrence of perimembranous VSD in 2 cousins (III 14, 13).


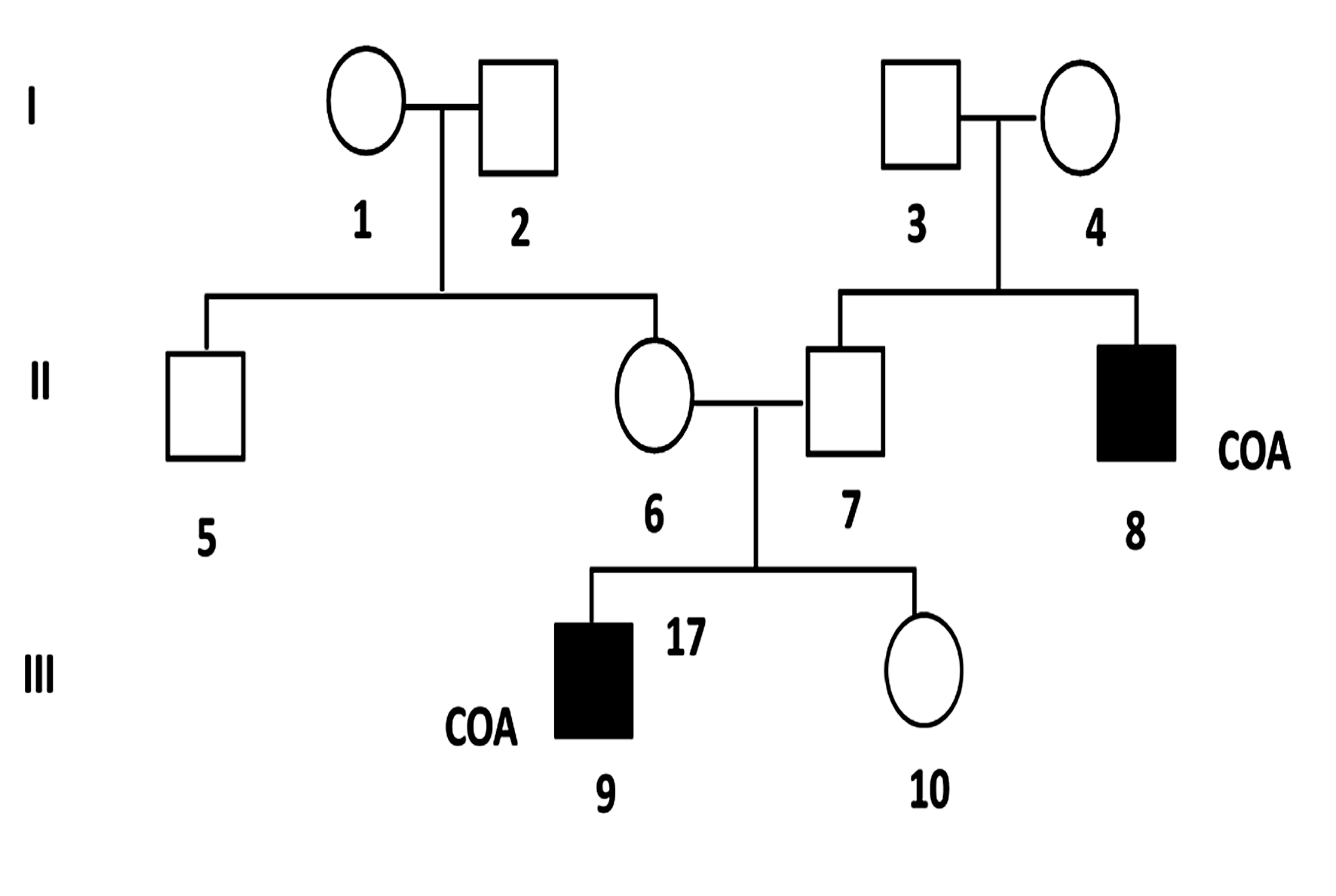


**Figure 5**: A Pedigree showing exact concordant recurrence of aortic coarctation in III.9 and his Uncle II.8.





**Figure 6:** A pedigree showing group concordant recurrence of CHD in sibling result of consanguineous marriage, Case 15: perimembranous VSD and secundum ASD and recurrence in brother with secundum ASD.





**Figure 7:** A Pedigree showing discordant recurrence of CHD of case III.13 with ASD and VSD, uncle had valvular aortic stenosis.





Figure 8: A pedigree showing pedigree showing discordant Recurrence of CHD among index case III.19 TOF and 2 cousins (17, 20) each with midmuscular VSD and perimembranous VSD
